# A roadmap for a patient-centred approach to Pompe disease management

**DOI:** 10.1007/s00415-026-13687-3

**Published:** 2026-02-14

**Authors:** Benedikt Schoser, Cristina Domínguez-González, Pascal Laforet, Andreas Hahn, Paul Gissen, Anna Kostera-Pruszczyk, Andreas Thalmeier, John Vissing

**Affiliations:** 1 Department of Neurology, Friedrich-Baur-Institute, Ludwig-Maximilians-University, Munich, Germany; 2 Hospital Universitario 12 de Octubre, imas12 Research Institute, Biomedical Network Research Centre on Rare Diseases (CIBERER), Instituto de Salud Carlos III, Madrid, Spain; 3 Neurology Department, Raymond-Poincaré Hospital, AP-HP, Nord-Est-Île-de-France Neuromuscular Reference Center, Garches, France; 4 Department of Pediatric Neurology, Justus-Liebig University, Giessen, Germany; 5 National Institute of Health Research Great Ormond Street Biomedical Research Centre, London, UK; 6 Department of Neurology, Medical University of Warsaw, ERN EURO NMD, Warsaw, Poland; 7 Pharmacy, LMU-Klinikum, Munich, Germany; 8 Copenhagen Neuromuscular Center, Rigshospitalet, University of Copenhagen, Copenhagen, Denmark

**Keywords:** Pompe disease, Rare genetic disorder, Gene therapy, Centres of excellence, Think-tank methodology, Multidisciplinary team

## Abstract

**Introduction:**

Pompe disease is a rare, progressive genetic disorder caused by pathogenic variants in the *GAA* gene. Emerging gene therapies offer the potential for long-term disease management, although logistical and clinical challenges demand specialised centres with defined protocols. A scientific steering committee of 8 European experts deliberated on the requirements for establishing gene therapy centres of excellence for Pompe disease.

**Methods:**

A modified think-tank approach was used to develop expert-based recommendations through qualitative research utilizing expert opinion methodology. Discussion topics were validated in an online kick-off meeting. Experts were assigned specific topics and tasked with generating content. Multiple online meetings facilitated expert presentations, discussions, and validation of recommendations for each topic.

**Results:**

Optimised patient management and timely access to treatment require accurate diagnosis and evaluation of Pompe disease. The committee recommended expanding newborn screening programs for infantile-onset Pompe disease and developing protocols for follow-up of presymptomatic late-onset Pompe disease. A specialised multidisciplinary team trained in Pompe disease and gene therapy should manage the patient journey. Pre-gene therapy assessments were recommended to mitigate risks. Patients should be hospitalized and continuously monitored during gene therapy infusions. After gene therapy, guidelines recommend corticosteroid immunosuppression, monitoring for adverse events (including hepatoxicity, myocarditis, thrombocytopenia, thrombotic microangiopathy, and hemophagocytic lymphohistiocytosis), and Pompe disease assessments (including motor functional assessments, magnetic resonance imaging of the muscles, and patient-reported outcomes). Centres of excellence require infrastructure with standard operating procedures for gene therapy products.

**Conclusions:**

Implementing gene therapy for Pompe disease requires a coordinated multidisciplinary effort to overcome gaps in knowledge, infrastructure, and patient management.

**Supplementary Information:**

The online version contains supplementary material available at 10.1007/s00415-026-13687-3.

## Introduction

Pompe disease, also known as glycogen storage disease type II (MIM#232300), is a rare autosomal recessive disorder caused by pathogenic biallelic variants in the *GAA* gene, which encodes the lysosomal enzyme acid alpha-1,4-glucosidase (GAA) and is crucial for the degradation of glycogen into glucose within lysosomes [1]. The deficiency of GAA activity leads to a progressive accumulation in the lysosome, which may rupture and release glycogen into the cytoplasm [2].

The pathogenesis of Pompe disease is driven by glycogen accumulation and the broader cellular dysfunction resulting from lysosomal and autophagic impairment [3]. Although the disease can affect multiple organs, the most severe clinical manifestations involve skeletal and cardiac muscles [4].

Pompe disease includes a spectrum of phenotypes, from the classic infantile form, which leads to mortality in early infancy, to the late-onset form, characterised by a more gradual progression primarily affecting skeletal and respiratory muscles in older children and adults [1, 5]. Infantile-onset Pompe disease (IOPD) is characterised by pathogenic variants that affect both alleles, resulting in a total or near-total deficiency of GAA enzyme activity (<1%) and an onset before 12 months of age. Clinically, IOPD is marked by generalised muscle weakness, severe hypotonia, hypertrophic cardiomyopathy, respiratory insufficiency, and cognitive decline. Without timely intervention, the disease is fatal, with most affected infants dying within the first year of life [6–9]. Late-onset Pompe disease (LOPD) is a heterogeneous disorder characterised by residual GAA activity levels ranging from 1 to 30% [1, 8]. LOPD can present at any age, typically after the first year of life, with variable progression rates and generally without significant cardiac involvement. LOPD clinically manifests as progressive weakness of the axial and limb-girdle muscles, often with early respiratory involvement, ultimately leading to substantial motor disability and respiratory failure [8, 10].

Estimates of Pompe disease prevalence have varied over time, influenced by newborn screening (NBS) initiatives and increased disease awareness. Epidemiological studies have consistently shown regional discrepancies due to variability in disease classification, differences in diagnostic approaches across NBS programs, and variations linked to consanguinity, race, and ethnic composition. Prevalence estimates vary widely across studies and countries, with frequencies reported as low as 1:297,387 in Japan and as high as 1:62,182 in Taiwan for IOPD and from 1:82,914 in Taiwan to 1:2628 in the US state of Washington, DC, for LOPD [11].

Enzyme replacement therapy (ERT) with recombinant human alpha-glucosidase (rhGAA) was approved by the United States Food and Drug Administration (FDA) and the European Medicines Agency (EMA) in 2006 for all patients with Pompe disease. Early initiation of the treatment has been linked to improved outcomes [12]. However, especially in patients with IOPD, responses to ERT can vary significantly, influenced by factors such as cross-reactive immunological material (CRIM) status, the development of anti-rhGAA immunoglobulin G antibodies, age at treatment initiation, ERT dosing, and the presence of severe cardiac involvement, invasive ventilation, or failure to thrive at baseline [13]. To mitigate the negative impact associated with elevated anti‑drug antibody levels, immunosuppressive strategies may be implemented [14, 15]. The development of next‑generation ERT, together with other emerging therapeutic modalities, has broadened the available options for managing Pompe disease, as extensively reviewed in recent literature [16–18]. Furthermore, the European Pompe Consortium has published expert recommendations detailing criteria for initiating, switching, or discontinuing ERT to guide clinical decision‑making [19].

Gene therapy is a novel approach to boost endogenous GAA production in affected cells. One of the key benefits of gene therapy is its ability to provide prolonged transgene expression following a single drug application, in contrast to the frequent infusions required for ERT. Adeno-associated viruses (AAVs) are the most promising vectors for therapeutic gene delivery in this context as they are non-pathogenic, can target a broad spectrum of tissues, and provide sustained gene expression with limited genome integration [20]. There are a few gene therapies in early clinical stages for Pompe disease, mainly in patients with LOPD. AAV8-MSP-hGAA (AT-845) is an AAV-based gene for LOPD that was initiated as a phase I/II clinical trial (FORTIS; NCT0417405) in October 2020 [20, 21]. Interim results from four participants over 2 years showed good tolerance, evidence of muscle vector transduction, and allowed three patients to stop ERT [22]. In addition, other gene therapy programs for Pompe disease are currently in clinical development, including the AAV9-mediated gene therapy GC301 for both IOPD [23] and LOPD (NCT06391736); the AAV gene therapy AB‑1009 for LOPD (PROGRESS‑GT; NCT07282847); ACTUS‑101 (NCT03533673) [24] and SPK‑3006 (RESOLUTE; NCT04093349), both liver‑directed AAV gene therapies, for LOPD; and the RNA interference–based therapy ABX1100 (NCT06109948).

Gene therapy has the potential to represent a breakthrough for this patient population. Still, only a limited number of centres possess the necessary expertise and resources to administer and manage this type of treatment [20]. There is no prior experience, established standard operating procedures (SOPs), or specialised centres dedicated to managing gene therapy for Pompe disease. However, experience from the administration of gene therapy medicinal products (GTMPs) for other conditions, such as spinal muscular atrophy (SMA) type 1 [25–27], haemophilia [28, 29], Duchenne muscular dystrophy (DMD) [30], and aromatic L-amino acid decarboxylase deficiency [31], serves as valuable references.

In this context, this report aims to define the requirements for establishing a centre of excellence or specialised unit for Pompe disease capable of incorporating gene therapy into patient management based on the expertise of a multinational scientific steering committee with extensive experience in managing Pompe disease and gene therapy, while acknowledging that these recommendations may require refinement pending the definitive therapeutic indications and regulatory requirements are clarified following potential approval.

## Methods

An exploratory online survey was conducted among thirteen members of the European Pompe Consortium (EPOC) to assess their clinical experience with gene therapy and their awareness of European, national, and local recommendations for gene therapy (Supplementary Appendix).

A multinational Scientific Steering Committee of eight experts was established based on their expertise in Pompe disease and on their clinical experience managing patients receiving gene therapies for neuromuscular and lysosomal disorders. The experts were six neurologists, one expert in lysosomal storage disorders, and one hospital pharmacist (Error! Reference source not found.). A chairperson led the committee.

**Fig. 1:**
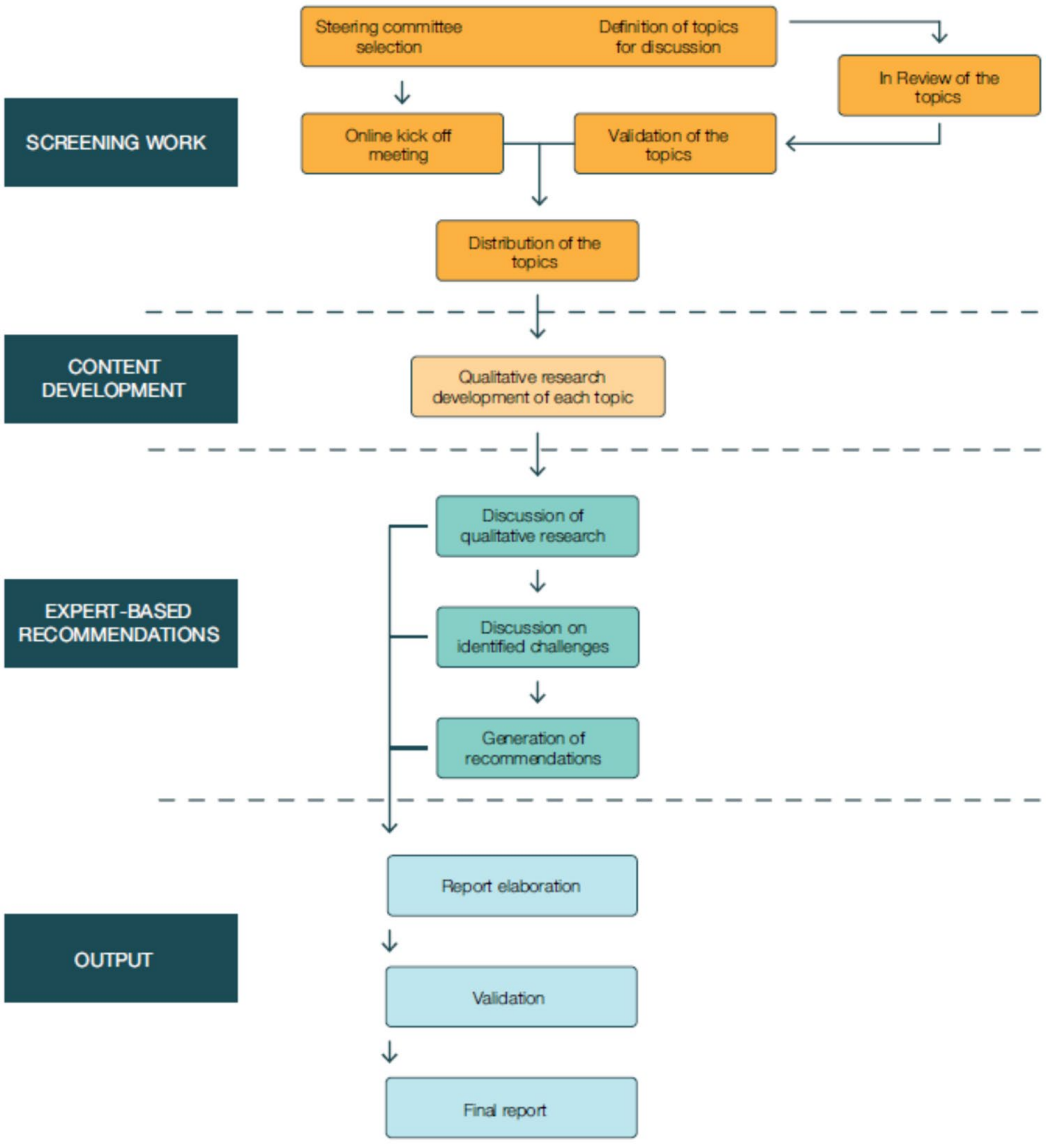
Methodological process

Expert-based recommendations were developed through qualitative research using a nominal group technique (or expert opinion). This modified *think-tank* methodology uses structured, facilitated meetings in which experts collaboratively gather insights on the topic through structured discussion [32]. An overview is shown in Fig. 1. The chairperson developed a list of candidate discussion topics focused on optimising the management of gene therapy within a centre of excellence for Pompe disease. During an online kick-off meeting, the Steering Scientific Committee validated the topics: (1) Patient diagnosis and evaluation; (2) Patient follow-up and support for patients and caregivers; (3) Multidisciplinary expertise and research and development initiatives; and (4) Infrastructure and resources for the handling, administration, and management of gene therapy treatments and products. Experts were then assigned to specific topics based on their areas of expertise. Each expert developed content for their assigned topic, including (1) a review of current state-of-the-art knowledge and evidence, (2) identification of key clinical practice challenges, and (3) proposed solutions to address these challenges, initiating group discussions to reach consensus-based recommendations. Following individual topic analyses, the chairperson and selected members of the Steering Committee conducted an initial review of the content via email. An online meeting facilitated expert presentations of findings on each topic, discussions of identified challenges, and individual recommendations. A collaborative consensus on each recommendation and its degree of support was achieved through facilitated discussion, ensuring that all expert opinions were considered. This consensus was documented, followed by the review and validation by each expert. The Steering Committee approved the final expert-based recommendation document.

## Results

### Patient diagnosis and evaluation

**Fig. 2:**
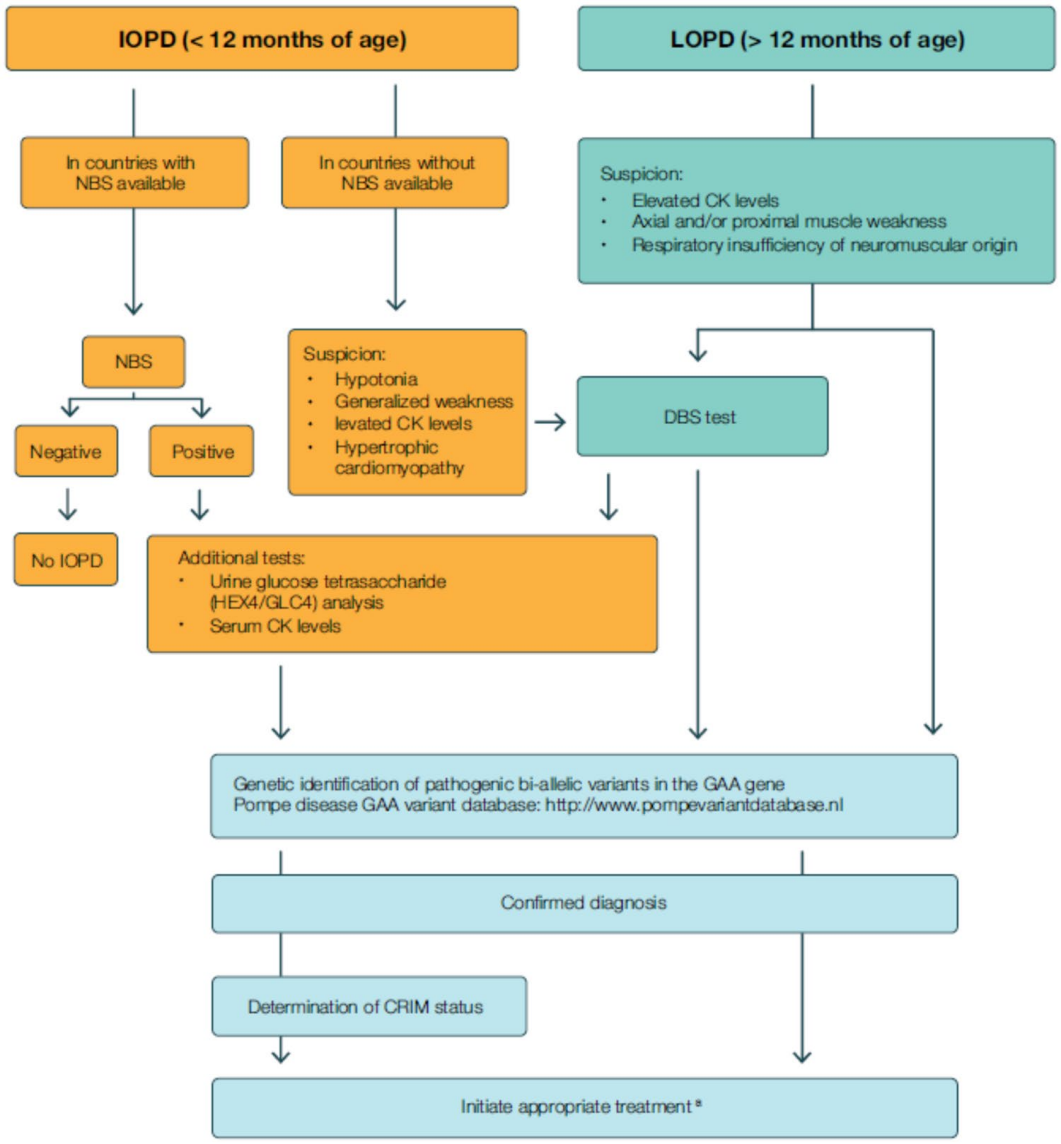
Pompe disease diagnosis. ^a^Appropriate treatment in IOPD includes ERT and immunomodulation. *CK* Creatine kinase; *CRIM* Cross-reactive immunological material; *DBS* Dried blood spot; *ERT* Enzyme replacement therapy; GAA Acid alpha-1,4-glucosidase; GLC4 Glucose tetrasaccharide; *IOPD* Infantile-onset Pompe disease; *LOPD* Late-onset Pompe disease; *NBS* Newborn screening

Delays in diagnosing Pompe disease are common due to its rarity, heterogeneity, and the lack of resources for a comprehensive evaluation of patients. Fig. 2 outlines the main steps for IOPD and LOPD diagnosis.

NBS for Pompe disease is recommended for all newborns, following its inclusion in the Recommended Uniform Screening Panel (RUSP) in 2015 and subsequent adoption by several countries [12, 13]. However, it is essential to note that NBS may encounter challenges, including the relatively high incidence of pseudodeficiencies, the detection of disease carriers, and variants of unknown significance [33]. Once a IOPD diagnosis is confirmed (Fig. 2), disease-modifying ERT should be initiated as soon as possible, as it is most effective when initiated early. CRIM status determines ERT-treatment response [12, 33] since CRIM-positive patients produce some endogenous GAA, enabling immunological tolerance of rhGAA and generally leading to more favourable ERT outcomes. Approximately 20% of patients with IOPD are CRIM-negative and lack detectable endogenous GAA, developing high-titre antibodies against exogenous rhGAA and reducing the long-term efficacy of ERT [34–38]. Immunomodulation can mitigate the immune response against rhGAA [14, 15].

Despite NBS can also identify LOPD, most LOPD cases are diagnosed when they are symptomatic. LOPD often manifests with proximal muscle weakness associated with respiratory insufficiency, although it can also be identified in a presymptomatic phase by elevated CK levels. Among presymptomatic individuals with LOPD identified by NBS, the unpredictable onset of symptoms demands a standardized and close longitudinal monitoring to ensure that treatment is initiated promptly after evidence of clinical pathology [39–41]. Evidence suggests that optimal response to ERT may occur when therapy is initiated at the earliest detectable signs of disease activity (skeletal or respiratory muscle weakness identified on clinical examination and/or spirometry) [5, 39]. The 2017 European consensus recommends follow‑up for presymptomatic individuals every six months during the first year after diagnosis and annually thereafter [5]. According to the Pompe Disease Newborn Screening Working Group, asymptomatic infants should be evaluated at 3 months of age and every 3 months in the first year, with continued evaluations every 3–12 months depending on clinical findings [39]. Thus, presymptomatic patients require careful clinical management and long-term monitoring. The dried blood spot (DBS) test is an effective screening tool, but diagnostic confirmation is always required (Fig. 2), including identification of pathogenic biallelic variants in the *GAA* gene [5, 41, 42]. The Pompe disease *GAA* variant database (http://www.pompevariantdatabase.nl) provides a curated, open-source resource listing of disease-associated variants and associated clinical phenotypes [43]. Importantly, no clear genotype-phenotype correlation has been established among patients with LOPD, and intrafamilial variability has been documented [44–47].

### Diagnostic tools

Several tools are used to accurately diagnose Pompe disease. GAA activity in Pompe disease is measured using different methods, substrates, and tissues (Table 1) [22, 48, 49]. Caution is warranted, as GAA testing can yield false results due to improper blood spotting, sampling errors, and environmental conditions (e.g., high temperatures during transport) [50–52]. Nevertheless, it can be performed using urinary glucose tetrasaccharide (GLC4) analysis, which helps identify IOPD in NBS programs [53, 54]. Concerning LOPD, the relationship between urine GLC4 levels and clinical severity has yet to be fully explored [55].

**Table 1:** Diagnostic tools

Diagnostic Tools – IOPD and LOPD				
GAA activity analysis	Methods: Fluorometry Tandem mass spectrometry	Substrates: Glycogen 4-methylumbelliferyl-α-D-glucoside (4MUG)	Tissues: Blood (DBS) *Does not differentiate IOPD/LOPD* [33] Leukocytes *Does not differentiate IOPD/LOPD* [33] Fibroblasts Muscle	Highlights: GAA activity is reduced in: Individuals with pseudodeficiency alleles Some carriers of the c.−32-13T>G variant [33, 65] It does not predict clinical outcomes for LOPD [66]
Urinary HEX4/GLG4	Method: UPLC-MS/MS assay [53]	Sensitivity: 98.5% Specificity: 92%	Highlights: Correlate with muscular glycogen content in quadriceps biopsies in IOPD [67] Not elevated in asymptomatic LOPD [55] Increased levels in the absence of the c.−32-13T>G variant [54]	
CK levels	Frequently > 2-5x the ULN in patients with Pompe disease [56]			
EMG	Can reveal a myopathic pattern with myotonic discharges in the paraspinal muscles or fibrillation potentials			
Muscle biopsy	When? Complementary tests yield inconclusive results		Marker: Acid phosphatase activity is the most sensitive histochemical marker	Result: Activity elevated in up to 82% [56]
Muscle MRI	Advantages [58]: High sensitivity Detecting fat replacement in muscle Lack of side effects Avoiding ionising radiation exposure		Highlights [59]: Most evidence is focused on patients with LOPD. In LOPD, MRI shows a consistent pattern of muscle involvement with progressive fatty replacement. Direct measurement of glycogen content through magnetic resonance spectroscopy represents a promising biomarker	
Comprehensive evaluation	Motor function assessments (*as manual muscle testing*) Functional motor scales Timed performance tests Respiratory evaluations Cardiac monitoring Cerebrovascular and aortic arc dilatation screening [63, 64]			

*4MUG* 4-methylumbelliferyl-α-D-glucoside; *CK* Creatine kinase; *DBS* Dried blood spot; *EMG* Electromyography; *GAA* Acid alpha-1,4-glucosidase; *GLG4* Glucose tetrasaccharide: *HEX4* Hexose tetrasaccharide; *IOPD* Infantile-onset Pompe disease; *LOPD* Late-onset Pompe disease; *MRI* Magnetic resonance imaging; *ULN* Upper limit normal; *UPLC-MS/MS* Ultraperformance liquid chromatography-tandem mass spectrometry

CK levels are often altered in Pompe disease, and their assessment can be helpful in the diagnostic pathway (Table 1). However, no correlation has been established between CK levels and the severity of muscle weakness [56]. Electromyography (EMG) is an unspecific and not very useful diagnostic tool for Pompe disease, and muscle biopsy can still play a role in diagnosing the disease, although vacuolar myopathy can be missed in approximately 30% of patients with LOPD (Table 1) [50, 56, 57]. Muscle magnetic resonance imaging (MRI) is a non-invasive tool that has become the preferred modality for muscle imaging, supporting the diagnosis of Pompe disease and enhancing patient follow-up [48, 58, 59]. Finally, a thorough evaluation of patients with Pompe disease should consider all potentially affected organs, including the respiratory, cardiac, and cerebrovascular systems [49, 59–64] (Table 1).

### Challenges and recommendations

**Fig. 3:**
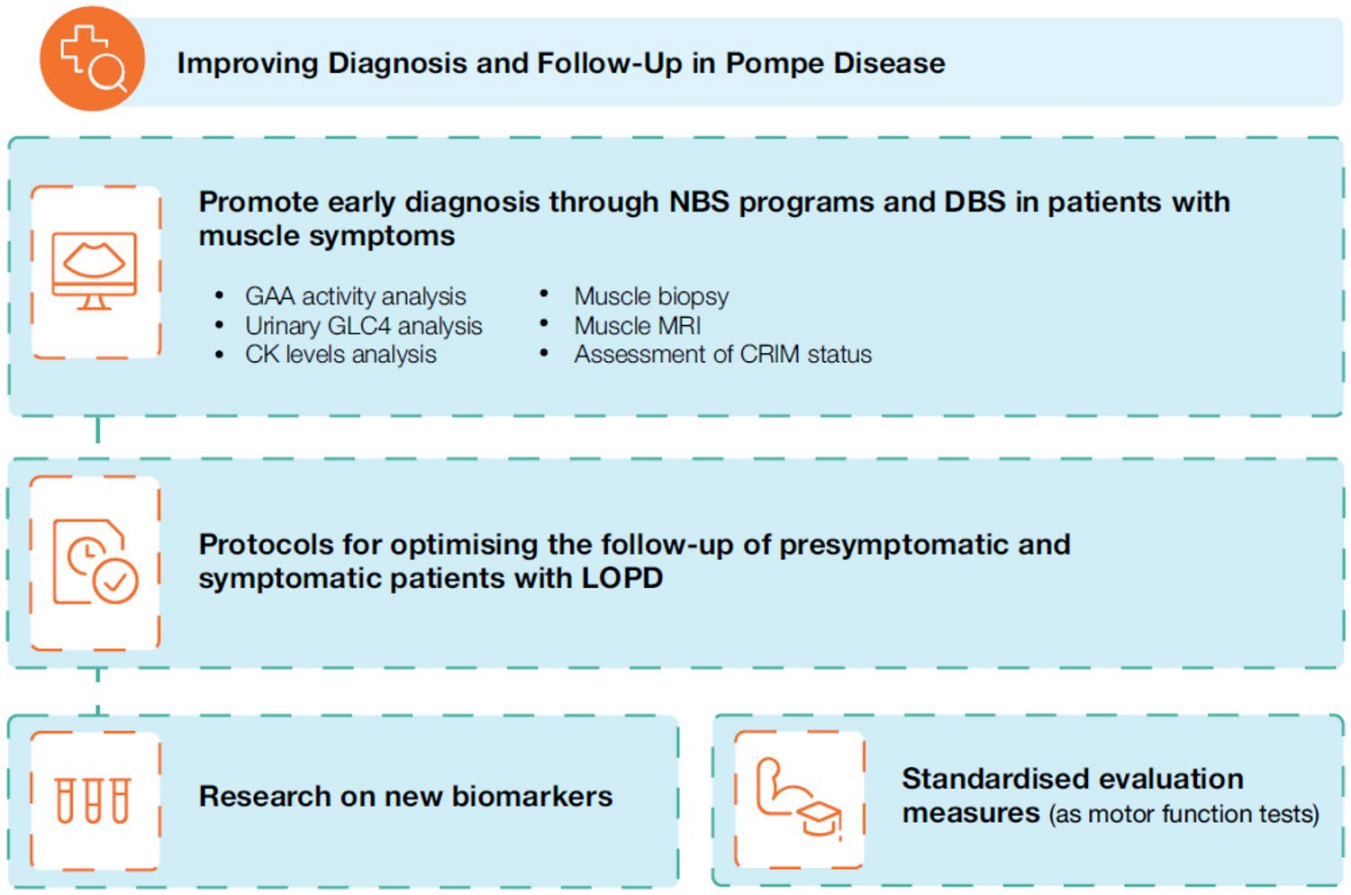
Diagnostic and evaluation recommendations for a centre of excellence for Pompe disease. *CK* Creatine kinase; *CRIM* Cross-reactive immunological material; *DBS* Dried blood spot; *GAA* Acid alpha-1,4-glucosidase; *GLC4* Glucose tetrasaccharide; *LOPD* Late-onset Pompe disease; *MRI* Magnetic resonance imaging; *NBS* Newborn screening

Improving diagnosis and evaluation is crucial for optimising patient management in Pompe disease. Fig. 3 outlines recommendations for a gene therapy centre of excellence for the disease. Early diagnosis remains a significant challenge and should be prioritized to ensure timely treatment initiation and effective disease management.

Experts recommended expanding, where feasible, NBS programs to accurately and rapidly detect IOPD cases and assess CRIM status. The implementation of comprehensive diagnostic protocols combining enzymatic assays and genetic testing is highlighted. To optimise the follow-up of presymptomatic patients with LOPD, experts recommend developing specific protocols for this subgroup.

Currently, no LOPD biomarkers are available that reliably predict clinical outcomes or guide treatment initiation decisions. The experts support further research to find and confirm reliable biomarkers, highlighting the direct measurement of glycogen content through magnetic resonance spectroscopy as a promising biomarker despite its technical challenges in implementation [68, 69].

There is also a need to standardise motor function tests that most accurately reflect the clinical status and progression of patients with Pompe disease.

After considering all the aforementioned challenges, experts recommended that the diagnosis and evaluation of patients with Pompe disease should be conducted at neuromuscular disease referral centres or lysosomal storage disorder centres, such as centres of excellence. These centres should also establish external collaborations when needed to enhance their capabilities.

### Patient follow-up and support for patients and caregivers

**Fig. 4:**
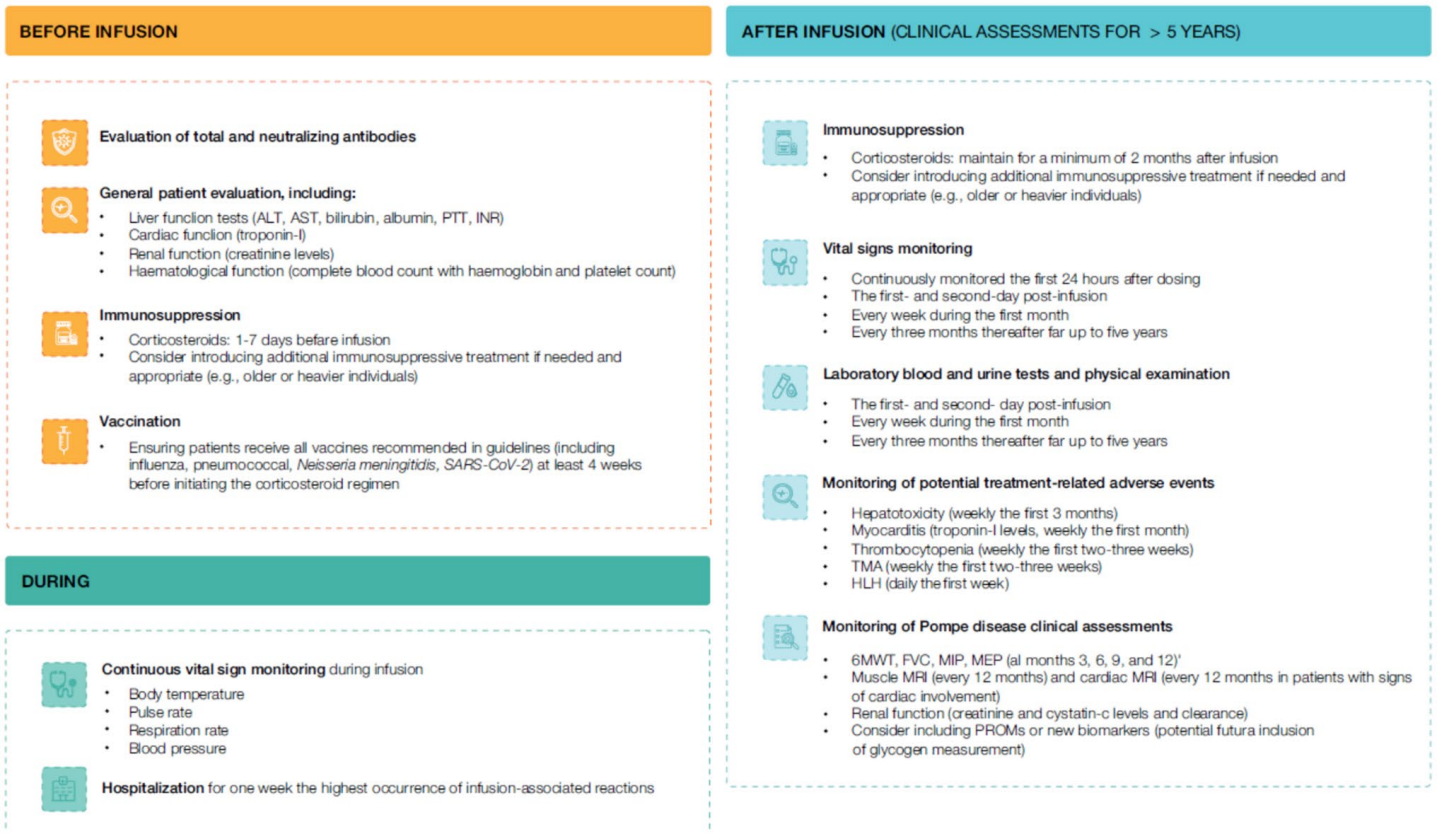
Recommendations for the follow-up of a patient before, during, and after the administration of gene therapy. ^a^From enzyme replacement therapy trials [83, 84]. *6MWT* 6-minute walk test; *ALT* Alanine aminotransferase; *AST* Aspartate aminotransferase; *FVC* Forced vital capacity; *HLH* Hemophagocytic Lymphohistiocytosis; *INR* International normalised ratio; *MEP* Maximum expiratory pressure; *MIP* Maximum inspiratory pressure; *MRI* Magnetic resonance imaging; *PROMs* Patient-reported outcome measures; *PTT* Partial thromboplastin time; *SARS-CoV-2* Severe acute respiratory syndrome coronavirus 2; *TMA* Thrombotic microangiopathy

A gene therapy centre of excellence for Pompe disease must have resources and detailed protocols to ensure a thorough patient evaluation before, during, and after therapy (Fig. 4), followed by long-term monitoring.

### Before gene therapy administration

Several aspects must be addressed before administering gene therapy to optimise patient outcomes and mitigate potential risks, as previously reported with GTMPs for other conditions, such as SMA or DMD [30, 70].

#### Evaluation of pre-existing anti-AAV antibodies

Screening patients for total antibodies against the vector is crucial to optimise transduction efficiency and minimise the risk of adverse events (AEs) related to immune responses against the viral vector [30]. Many adults may have pre-existing immunity to AAV due to environmental exposure to naturally occurring AAV [71, 72], and even low titres of neutralising antibodies can affect the transduction of the gene of interest [73, 74]. Consequently, all potential candidates for AAV gene therapy products should be assessed for total or neutralizing antibodies to AAV.

#### General patient evaluation

A comprehensive pre-infusion evaluation should determine whether the patient is suitable for gene therapy, minimising risks and improving monitoring and therapeutic outcomes (Fig. 3) [30]. Pre-treatment evaluations comprise hepatic, renal, haematological, and cardiac functions [70].

#### Corticosteroid immunosuppression

Immunomodulation with corticosteroids is recommended before and after gene therapy administration to mitigate the immune response to the AAV capsid (Fig. 3) [25, 30, 70].

#### Vaccination

Patients must receive all vaccines recommended in current guidelines before receiving the gene therapy (Fig. 4) [25, 30]. However, concurrent use of corticosteroids may reduce vaccine efficacy, and several GTMPs protocols adapt vaccination schedules accordingly [30]. Pompe disease is recognised as a risk factor for severe COVID-19, and vaccination for all patients is strongly recommended according to local guidelines and the current epidemiological situation, given its demonstrated safety and effectiveness in preventing severe COVID-19 [75].

### During gene therapy administration

During gene therapy administration, it is recommended to continuously monitor vital signs, including body temperature, axillary temperature before and after dosing, pulse rate, respiration rate, and blood pressure during the infusion [25].

### After gene therapy administration

Following infusion, patients should ideally remain in the hospital for several days to closely monitor potential treatment-related AEs (Fig. 4). In clinical trials, biochemical parameters, biomarkers, and immunological, physical, and pulmonary assessments were monitored for up to two years to assess safety and efficacy [76]. The main AEs and recommended monitoring with other GTMPs using AAV vectors are outlined in Table 2.

**Table 2:** Main adverse events of gene therapy with an AAV vector

Adverse Event	Description	Recommended short-term monitoring (first 30 days)	Recommended long-term monitoring (from day 30)
Hepatotoxicity	Elevated aminotransferase levels, generally manifested by increased ALT and/or AST levels, may indicate immune-mediated hepatotoxicity and potentially lead to severe liver injury and acute liver failure [70].	Weekly monitoring with adjustments to the corticosteroid regimen if clinically indicated [30, 70]. In clinical trials of onasemnogene abeparvovec, cases of severe adverse events (SAEs) related to hepatotoxicity, such as elevated serum aminotransferase levels, were managed with prednisolone treatment [76].	Weekly monitoring with adjustments to the corticosteroid regimen if clinically indicated. Regular monitoring of liver function until the results return to normal [30, 70]. In clinical trials of onasemnogene abeparvovec, cases of SAEs related to hepatotoxicity, such as elevated serum aminotransferase levels, were treated with prednisolone [76].
Myocarditis	Signs of myocarditis include elevated troponin I levels, decreased ejection fraction, focal wall-motion abnormalities on echocardiography, and increased T2-weighted signal on cardiac magnetic resonance imaging (MRI)[70, 77].	Weekly troponin I monitoring and bi-weekly echocardiogram monitoring [30, 70].	Continued monitoring if clinically indicated [30].
Thrombocytopenia	Transient decreases in platelet counts, some of which met the criteria for thrombocytopenia. The lowest platelet count often occurred during the first week after the infusion [30, 70].	Weekly monitoring for the first two/three weeks [30, 70].	Continued monitoring if clinically indicated [70].
Thrombotic microangiopathy (TMA)	TMA, an acute and life-threatening condition, is characterised by thrombocytopenia and microangiopathic haemolytic anaemia and may occur following infusion with gene therapy [70].	Platelet counts should be closely monitored during the first two/three weeks following infusion, with ongoing monitoring thereafter. In cases of thrombocytopenia, further evaluation, including diagnostic tests for haemolytic anaemia and renal dysfunction, should be promptly conducted [70].	Continued monitoring if clinically indicated [70].
Hemophagocytic Lymphohistiocytosis (HLH)	HLH, a rare, life-threatening hyperinflammatory syndrome, is characterized by fever, markedly elevated ferritin, and cytopenia. This immune dysregulation might lead to multi-organ damage. HLH has been rarely reported following high-dose AAV gene therapy [78].	Daily monitoring of ferritin, fever, and cytopenia during the first week following infusion. Continuous monitoring should be maintained throughout the first 30 days, with daily assessments if liver transaminase levels are elevated [78].	

*AAV* Adeno-associated virus; *ALT* Alanine aminotransferase; *AST* Aspartate aminotransferase; *HLH* Hemophagocytic Lymphohistiocytosis; *MRI* Magnetic resonance imaging; *SAE* Severe adverse events; *TMA* Thrombotic microangiopathy

### Support for patients and caregivers

Caregivers play a crucial role and must be well-informed throughout the process. They should understand the corticosteroid regimen, including the importance of adhering to the prescribed dosage and contacting a healthcare provider immediately if the patient misses a dose or vomits shortly after taking one. They should also be aware of the vaccination schedule to ensure timely and appropriate immunisations, the primary risks associated with gene therapy, including its signs and symptoms, the requirement to test patients for the presence of AAV antibodies, and the need for increased vigilance to prevent, monitor, and manage infections before and after AAV infusion [70, 76].

### Challenges and recommendations

Developing robust follow-up and monitoring protocols and recommendations for gene therapy in Pompe disease relies on leveraging experience from similar diseases. The expert recommendations are outlined in Fig. 4. It should be acknowledged that recommendations may vary depending on the specific gene therapy and the outcomes of ongoing clinical trials. Further development of these recommendations is needed to include detailed guidance on monitoring disease status following gene therapy, which is crucial for informing decisions about discontinuing or initiating ERT. More advanced immunosuppressive strategies, including the addition of non-steroidal immunosuppressants, should also be considered.

Treatment approaches and monitoring might be tailored to the phenotype. For example, IOPD may require a more intensive strategy with close monitoring due to its rapid progression and severity, whereas managing patients with LOPD may involve less frequent reviews tailored to individual needs, unless there is a significant clinical decline [79]. Monitoring neurological involvement is also important, and cranial MRI has been mainly recommended in IOPD, but also for patients with LOPD. Additionally, formal cognitive assessments should be considered when there are clinical concerns or if progressive leukodystrophy is detected [79, 80]. Furthermore, emerging biomarkers, such as plasma glial fibrillary acidic protein (GFAP) and neurofilament light chain (NfL), are being studied for their potential diagnostic value in evaluating CNS involvement [81, 82].

In addition, experts emphasised the need to prepare for the future expansion of gene therapy availability, advocating for sufficient centres of excellence for Pompe disease in each country to ensure patients receive follow-up care close to home. In countries such as France, Germany, and Poland, no fewer than 10 specialised centres care for patients with SMA. Experts considered this range of centres appropriate for follow-up and treatment administration for patients with Pompe disease.

Moreover, improved home monitoring of patients in real-world settings could facilitate the evaluation of the benefits of gene therapy and their impact on quality of life. Thus, experts recommend developing digital applications and wearable or connected devices to continuously monitor movement speed, activity levels, gait patterns, and respiratory function, providing valuable data to optimise patient care and outcomes.

### Multidisciplinary expertise and research and development initiatives

A specialised multidisciplinary team in Pompe disease and gene therapy is essential to adequately manage the entire patient journey within a centre of excellence. Evidence from similar conditions can guide the establishment of the optimal team composition [85]. In addition, research and development initiatives would generate real-world evidence to advance scientific knowledge and guide and improve future treatment protocols.

### Challenges and recommendations

#### Multidisciplinary team

**Fig. 5:**
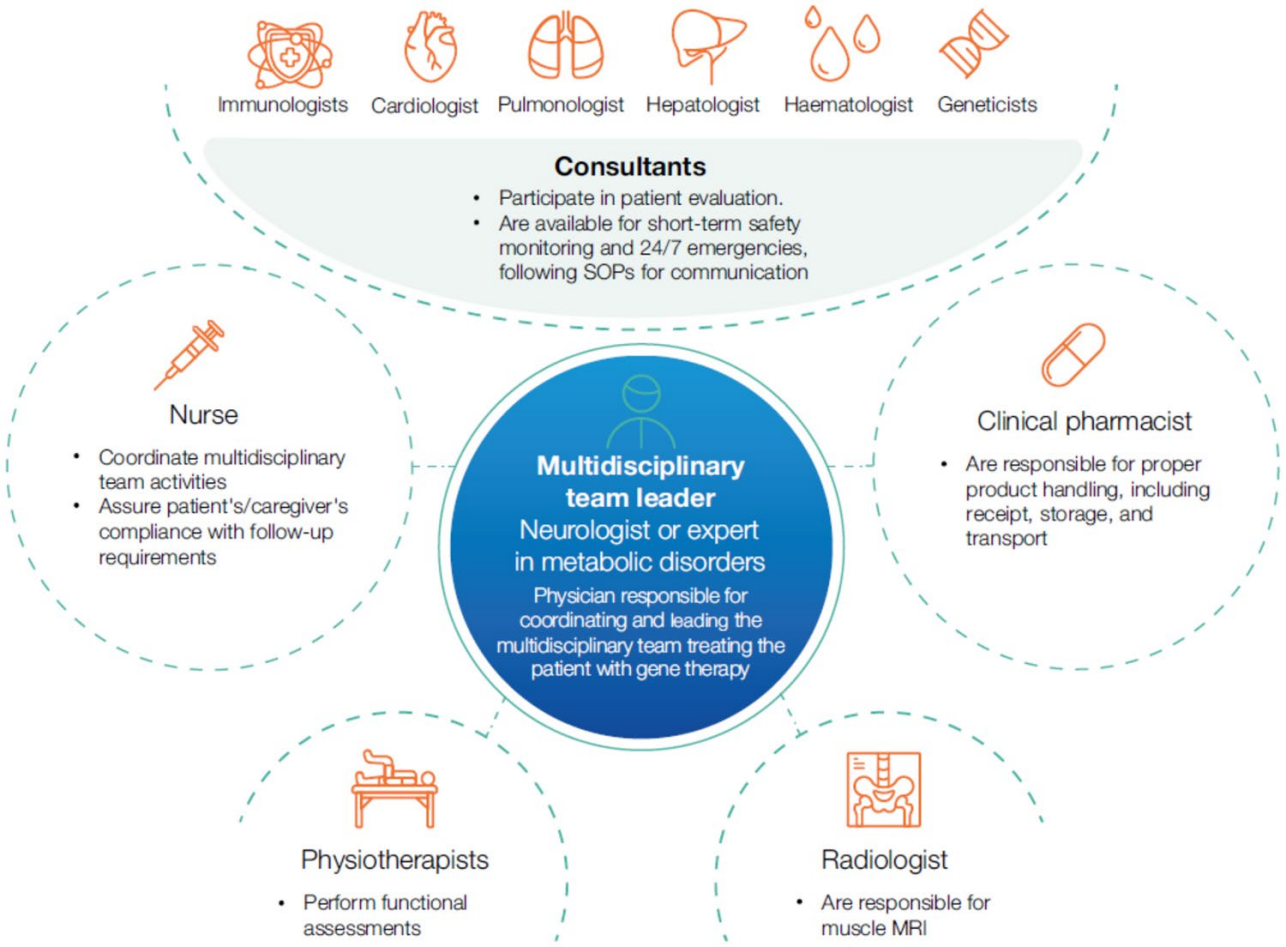
Multidisciplinary team profiles recommended by the experts for a gene therapy centre of excellence for Pompe disease. *MRI* magnetic resonance imaging; *SOP* standard operating procedure

Multidisciplinary team profiles recommended by the experts for a centre of excellence for Pompe disease are presented in Fig. 5.

An appropriate team leader would be a neurologist, a paediatric neurologist, or a clinician with expertise in metabolic disorders, who would coordinate and guide all experts involved in the patient’s diagnosis, assessment, and gene therapy treatment. Consulting specialists, including cardiologists, pulmonologists, hepatologists, geneticists, immunologists, and haematologists, could collaborate in pre-treatment assessment and post-treatment monitoring, including managing the immunosuppression of patients. All specialists should operate within well-defined SOPs. In addition, a specialised nursing team would be necessary to coordinate multidisciplinary activities, maintain direct communication with patients and caregivers, and educate patients and families about the disease and its management to ensure adherence to follow-up requirements. Clinical pharmacists would be essential for handling gene therapy products, including receiving, storing, and transporting them. Finally, radiologists would be responsible for muscle MRI assessments, and physiotherapists would be responsible for functional evaluations.

Regularly scheduled structured committee meetings would allow effective coordination of the multidisciplinary team and individual patient status updates.

Establishing an online European community with experts supporting healthcare professionals in managing gene therapy for Pompe disease would be valuable. The European Network of Reference Centres for Neuromuscular Diseases (ERN EURO-NMD), the European Academy of Neurology (EAN), and the World Muscle Society (WMS) could facilitate this community, promoting cross-border collaboration and best practices in clinical care.

Multidisciplinary team members should systematically document their expertise, qualifications, and prior experience, including involvement in administering gene therapy for other indications in clinical trials or routine care.

Developing educational programs with harmonised objectives would build and standardise disease-specific knowledge and expertise across Europe, allowing professionals to stay up to date with the latest advancements in gene therapy applications. Such programs should include formalised curricula in various formats, including webinars, presential workshops, accredited training, local and national study days, clinical observation opportunities, reading materials, and e-learning modules.

#### Research and development

Multidisciplinary teams are recommended to execute research and development initiatives, including systematically collecting data by participating in professionally curated databases within each country's specific resources and logistical constraints, thereby generating solid real-world evidence to build knowledge and improve treatment protocols.

### Infrastructure and resources for gene therapy

**Fig. 6:**
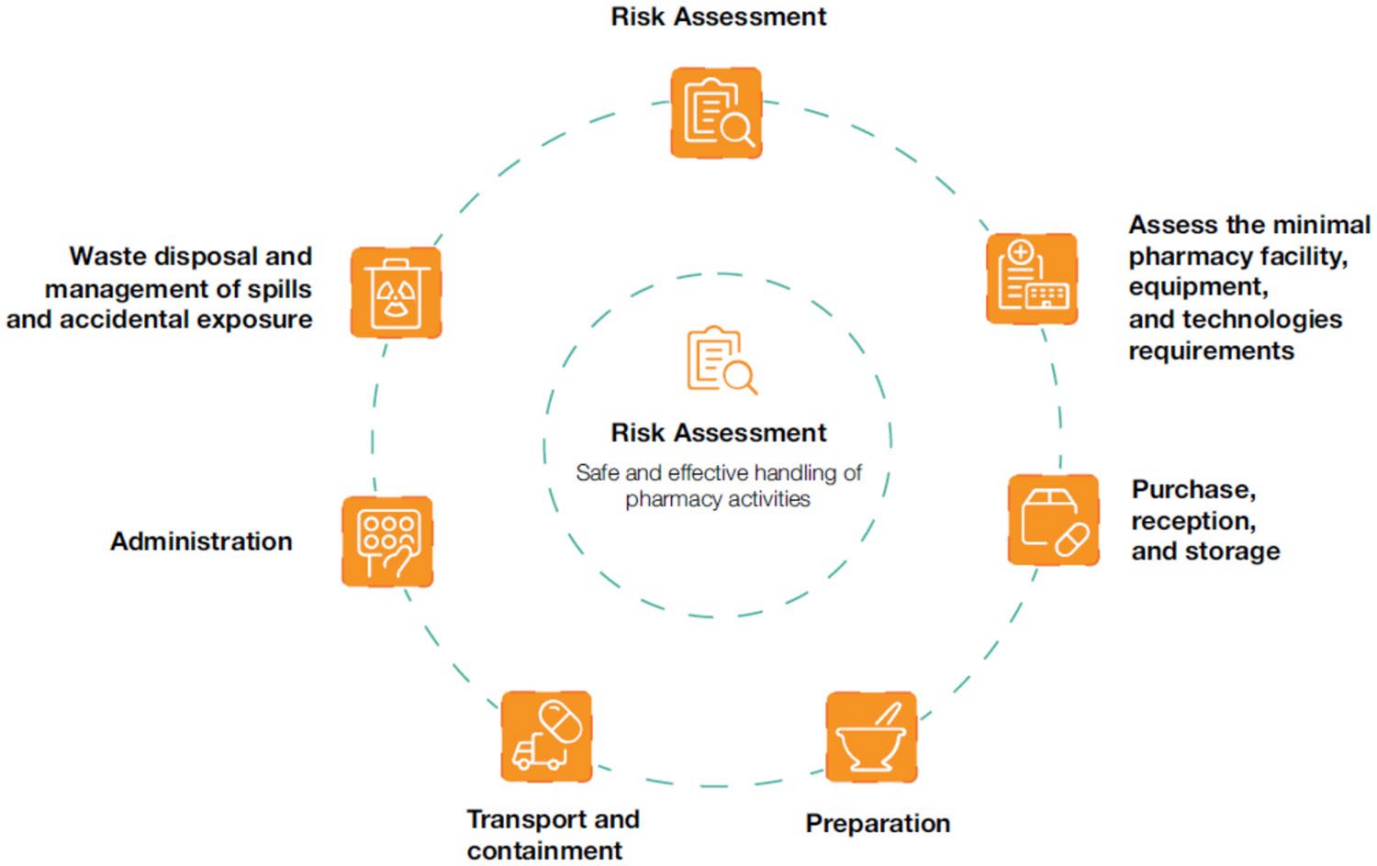
Key considerations for developing protocols for the administration of gene therapy

The use of gene therapy is expanding rapidly, with new GTMPs being approved each year. However, up-to-date guidelines and SOPs are needed to ensure the safe and efficient handling of GTMPs throughout the entire treatment process [86]. Existing SOPs for intravenously administered licensed products can inform the development of SOPs for gene therapy in Pompe disease [86]. Key aspects of developing gene therapy protocols are shown in Fig. 6 and Table 3 and are further discussed below.

**Table 3:** Checklist for the utilisation of infrastructure and resources for gene therapy

Risk assessment	
1.	Gene therapy vector details: plasmid map, transgene, additional sequences, and manufacturing process.
2.	Biosafety risk group classification of the GTMP and potential hazards and risks to human health.
3.	Patients involved and route of delivery.
4.	Facilities for: Reception of GTMP prior to administration. Storage of GTMP prior to administration. Infusion of GTMP. Immediate post-infusion care. Continuation of post-infusion care. Disposal of infusion materials and waste.
5.	Responsible person for GTMP preparation for infusion.
6.	Qualification and training relevant to the administration and handling procedures and responsible person for ensuring compliance.
7.	Need for specific handling of the GTMP and whether segregation of patients is required (e.g. immunodeficient patients on the ward, family members including siblings).
8.	Any possible interactions between GTMP and other concurrent medications.
9.	Any concerns regarding GTMP shedding in body fluids after patient discharge.
10.	Specific hazards identification: Safe transport, storage, and preparation. Concerns about inoculation or exposure to body fluids of healthcare workers.

Minimal pharmacy facility, equipment, and technology requirements	
11.	Pharmacy personal protective equipment depending on the activity performed, including disposable lab coats, gowns or aprons, safety glasses or goggles, gloves, and splash protection (i.e. face masks) [87].
12.	Qualified ultra-low temperature freezers for drug storage with 24-h calibrated temperature monitoring device [31, 87].
13.	Biological safety cabinet Class II type B or pharmaceutical grade isolator compliant with European standard EN 12469:2000 (recommendations for risk group 1 and risk group 2 GTMPs) [86].
14.	Spill kits for the decontamination of areas after drug spills [88].
15.	Biohazard-labelled leak-proof containers for transport around the institution [89].
16.	Virucidal agents appropriate to the GTMP (enveloped or capsid vector) [87].

Purchase, reception, and storage	
17.	Pharmacy personnel should wear appropriate personal protective equipment when unpacking GTMPs in case of transport-related damage [86].
18.	Store the GTMP in a designated area (fridge or freezer) according to the specified storage conditions [89].
19.	Display a biohazard warning sign on the storage unit, the entrance to the storage room, and the cleanroom where the product is prepared [90].
20.	Control the storage temperatures using a calibrated temperature-monitoring system [87, 89].

Preparation	
21.	Prepare, if possible, the GTMP in a Class II Type B biosafety cabinet or within a pharmaceutical-grade isolator, with background cleanroom standards complying with local regulations [86].
22.	Apply the stability data outline in the SmPC or clinical documents (if the GTMP is aseptically prepared in a grade A cleanroom environment) [91].
23.	All preparation steps should be supervised by a second individual [92].

Transport and containment	
24.	Transport GTMPs in a double-enclosed system (i.e. a plastic bag and a leak-proof biohazard-labelled container) [89].
25.	Transport personnel should be explicitly trained in GTMPs safe handling [89].
26.	Adhering to stability data and temperature requirements according to the SmPC or clinical trial documentation is essential [86].
27.	GTMP waste should be managed through incineration, autoclaving, or heat inactivation [86].
28.	Materials used during administration must be disposed of under careful regulations [87, 89].
29.	Universal precautions with patent material must be taken to ensure the safety of healthcare personnel and family members [90].

Administration	
30.	Patients may need to be isolated during and after the administration [86].
31.	Administration of the GTMP within the timeframe outlined in the SmPC, with the dose ideally being used immediately without storage in a second location [86].
32.	Coordination between clinical staff and the hospital pharmacy is crucial to ensure timely reconstitution and administration of the GTMP [86].
33.	Guide patients and caregivers about precautions to take at home [86].

Waste disposal and management of spills and accidental exposure	
34.	Availability of a spill management kit, along with an appropriate decontaminant, in all areas where a GTMP is handled [89].
35.	Seek medical attention and report the incident to the occupational health department, biosafety or infection control officer, and/or incident-report group, following established local protocols [86].

Risk assessment adapted from the Great Ormond Street Hospital in London, UK protocols

*GTMP* Gene therapy medicinal products; *SmPC* Summary of Product Characteristics

### Risk assessment

A thorough risk assessment must be conducted for new GTMPs in a hospital setting, primarily based on the EMA Summary of Product Characteristics (SmPC) and clinical trial documents. GTMPs should receive appropriate containment measures based on the risk assessment, which considers factors such as replication competence, potential for genome integration, and transgene toxicity. For instance, GTMPs classified as Risk Group 1 require containment under biosafety level 1 [86, 87]. All AAV serotypes fall under Risk Group 1 since they are non-infectious and not associated with disease in healthy adult humans, representing the lowest risk level [25].

According to protocols from hospitals with experience in GTMPs, such as the Great Ormond Street Hospital in London, UK, the clinical team lead should conduct the risk assessment, which should be reviewed and approved by a genetically modified organism (GMO) committee from the hospital, including pharmacists, clinical laboratory leads, hospital infection control leads, scientists with expertise in gene therapy viral vectors, and clinicians experienced in leading gene therapy clinical trials. Key elements for risk assessment are shown in Table 3.

### Minimal pharmacy facility, equipment, and technology requirements

Centres administering GTMPs should meet the minimum standards for pharmacy facilities, equipment, and technologies, as outlined in Table 3.

### Purchase, reception, and storage

Pharmacists are typically responsible for purchasing, receiving, and storing GTMPs [86]. Particular attention must be paid to financial clearance and purchasing procedures, given the significant economic costs associated with these therapies [93]. Upon delivery, personnel should wear the appropriate equipment, and GTMPs should be stored appropriately, as indicated in Table 3 [86, 87, 89, 90].

### Preparation

*In vivo* GTMPs should, whenever possible, be prepared within pharmacy departments to reduce the risks of environmental contamination, microbial exposure, and medication errors [88]. It is recommended that preparation is conducted Class II Type B biosafety conditions (Table 3) [86] and that a second individual verify all critical preparation steps [92]. If the GTMP is aseptically prepared in a grade A cleanroom environment, the stability data outlined in the SmPC or clinical trial documents can be applied. Otherwise, the product must be administered immediately after preparation [86].

Safety protocols, such as those outlined in the United States Pharmacopeia (USP) General Chapter Hazardous Drugs—Handling in Healthcare Settings, provide standards for handling hazardous drugs to protect healthcare staff, patients, and the environment [90]. Moreover, according to the PAN UK Pharmacy Working Group for Advanced Therapy Medicinal Products, process maps are recommended as an effective tool for developing aseptic preparation workflows [88]. A risk assessment of the aseptic preparation process should cover the transfer processes in and out of the cleanroom and isolator, reconstitution procedures, required consumables, disposal of used materials, format of final packaging, labelling, stability and storage requirements, development of worksheets and SOPs, and actions in the event of spillage.

### Transport and containment

Prepared GTMPs should be transported in a double-enclosed system and by trained personnel (Table 3) [89]. Any remaining GTMP waste should be disposed of, ensuring appropriate decontamination [86]. Non-disposable items should undergo decontamination procedures on the ward [87, 93]. Furthermore, strict precautions must be observed when handling and disposing of needles and other sharps to prevent accidental injuries or contamination [86]. After GTMP administration, universal precautions must be taken with patient material, ensuring the safety of healthcare personnel and family members based on available data on viral shedding for the specific therapy [90].

### Administration

GTMP administration is typically the responsibility of the treating physicians, with support from the nursing team. Depending on the extent of viral shedding, patients may need to be isolated during and after administration (Table 3). Before administration, it is critical to double-check the product, prepare the administration site, and ensure the patient is ready to receive the treatment promptly after preparation. The GTMP should be administered within the timeframe outlined in the SmPC, preferably immediately after preparation. Thus, coordination between clinical staff and the hospital pharmacy is crucial. Moreover, patients and caregivers must be guided on necessary precautions at home, as viral shedding could lead to antibody development in family members with the same condition [86]. For example, with onasemnogene abeparvovec, caregivers are instructed to wear protective gloves when handling the patient's faeces and to practice rigorous hand hygiene for approximately 60 days after the infusion [25].

### Management of spills and accidental exposure

A spill management kit and an appropriate decontaminant should be available in all areas where a GTMP is handled to ensure safety and containment of any unintentional exposure [89]. The recommended contents of the spill kit include two disposable gowns or arm covers, four pairs of gloves, two masks, two aprons, two pairs of goggles, four pairs of disposable shoe covers, two disinfectant sachets or pre-prepared decontaminants, absorbent paper towels, two disposable forceps, two biohazard incineration bags, the emergency contact number, and a copy of the spillage procedure [86].

In the event of accidental exposure to viral vectors, seek medical attention and report the incident to the occupational health department, the biosafety or infection control officer, and/or the incident-report group, following established local protocols. Inhalation, skin exposure, eye or mucous membrane splash, and needlestick injury should be addressed promptly [86].

### Challenges and recommendations

Experts recognised that enhanced expertise is needed to handle GTMPs and conduct vector risk assessments, which challenge the implementation of centres of excellence for Pompe disease. Table 3 collects the minimal requirements and procedures for implementing such a centre**.** Some specific aspects are discussed below.

#### Infrastructure

Only a few hospitals meet the minimal requirements for pharmacy facilities, equipment, and technologies necessary to handle GTMPs safely. For instance, many pharmacies lack dedicated biosafety units, Class II biological safety cabinets, or ultra-low temperature freezers. Experts recommend that centres of excellence for Pompe disease proactively assess their infrastructure and equipment to ensure compliance with national regulations and to be adequately prepared to administer GTMPs.

#### Training

Staff may have concerns about handling GTMPs, often due to the potential for these costly therapies to spill and the risk of accidental self-exposure. Experts advise that centres of excellence for Pompe disease implement comprehensive training programs for all staff involved in GTMP management, including personnel responsible for cleaning patient rooms, biosafety laboratories, and transport staff, who should also receive training in spill management protocols. Importantly, these therapies are typically approved medications, likely classified as Risk Group 1, posing minimal harm to humans. Training will promote a safer working environment and improve staff confidence in handling GTMPs. Furthermore, patient and family education with explicit, easily understandable materials in the appropriate languages should ensure compliance with safety measures.

#### Guidelines and SOPs

There are no mandatory European guidelines governing the handling of GTMPs, and national regulations vary significantly across European countries. Experts recommend that each centre of excellence develop comprehensive institutional SOPs and policies that address all aspects of GTMP application, adhere to the SmPC and clinical trial documents, and include the critical points outlined in Table 3. Additionally, a thorough risk assessment for each GTMP is essential before its initial administration. Experts strongly advise starting this process as soon as possible, as administrative procedures can be time-consuming and vary by country.

#### Prescribing systems

Adopting new dosing units, such as vector genomes, must be consistently reflected across electronic prescribing systems and medical records. Consequently, experts recommend that centres of excellence for Pompe disease evaluate whether these new units are integrated into their existing systems to ensure accurate dosing and effective management of gene therapy treatments.

## Conclusion

Gene therapy for Pompe disease represents a paradigm shift in the management of this rare genetic disorder, offering the potential for long-term, transformative benefits. However, gene therapy also presents significant logistical, clinical, and organizational challenges that could be managed by establishing centers of excellence. Centers of excellence should bring together multidisciplinary teams with expertise in Pompe disease and gene therapy to provide comprehensive, patient-focused care. Key priorities include early and accurate diagnosis of patients through NBS programs, DBS screening of patients with muscle symptoms, and the use of all available diagnostic tools, and implementing optimised follow-up protocols pre- and post-gene therapy. Continuous monitoring for AEs, particularly during the acute and long-term post-infusion periods, would ensure patient safety and maximise therapeutic outcomes. The complexities of gene therapy administration demand specialised infrastructure and resources, including upgrading pharmacy facilities, availability of biosafety-level equipment, and developing specific SOPs for gene therapy. Additionally, training programs for healthcare professionals and caregivers would enhance their capacity and confidence in managing this advanced therapy.

Research and development should be fundamental in centres of excellence, contributing to refining treatment protocols and identifying novel biomarkers to guide clinical decision-making. European collaborative networks can harmonise standards, promote best practices, and establish a unified framework for gene therapy delivery.

In summary, implementing gene therapy for Pompe disease requires a coordinated effort to overcome existing gaps in knowledge, infrastructure, and care delivery. Overcoming these challenges would allow healthcare systems to fully realize gene therapy's potential, leading to better patient outcomes and setting a new standard for managing rare diseases.

## Supplementary Information

Below is the link to the electronic supplementary material.

Supplementary file1 (DOCX 75 KB)

## Data Availability

Data availability is not applicable, as this expert opinion article presents recommendations derived from a collaborative consensus among experts.
