# Supplementary material for: A roadmap for a patient-centred approach to Pompe disease management
Source: J Neurol. 2026 Feb 14;273(2):145. doi: 10.1007/s00415-026-13687-3 (PMC12906549; doi:10.1007/s00415-026-13687-3)
Supplement: Supplementary file 1 — Supplementary file1 (DOCX 75 KB) [file 415_2026_13687_MOESM1_ESM.docx]

# Supplementary information

Supplementary Table 1 Steering Scientific Committee

| **Expert** | **Country** | **Specialist** |
| --- | --- | --- |
| ***Chairperson*** | | |
| Prof. Benedikt Schoser | Germany | Neurologist |
| ***Steering Committee members*** | | |
| Prof. Paul Gissen | United Kingdom | Expert in lysosomal storage disorders |
| Dr. Cristina Domínguez | Spain | Neurologist |
| Prof. Pascal Laforet | France | Neurologist |
| Prof. John Vissing | Denmark | Neurologist |
| Prof. Anna Kostera-Pruszczyk | Poland | Neurologist and paediatric neurologist |
| Prof. Andreas Hahn | Germany | Paediatric neurologist |
| Dr. Andreas Thalmeier | Germany | Hospital pharmacist |

## Supplementary Appendix 1 Survey

### Healthcare provider information

Germany had the largest representation of healthcare providers (HCPs) (23%) (**Supplementary Appendix Table 1**). Most participants (69.2%) were neurologists, followed by metabolic specialists (15.4%). Additionally, over half of the respondents (61.5%) were affiliated with centres with gene therapy experience, whereas 38.5% worked in centres without such expertise.

Supplementary Appendix Table 1 Characteristics of HCPs

| **Characteristics** | **Nº of HCPs (%) (n=13)** |
| --- | --- |
| **Country** |  |
| Germany | 3 (23%) |
| Luxembourg | 1 (7.7%) |
| Hungary | 1 (7.7%) |
| France | 1 (7.7%) |
| Denmark | 1 (7.7%) |
| Italy | 1 (7.7%) |
| Austria | 1 (7.7%) |
| Greece | 1 (7.7%) |
| Poland | 1 (7.7%) |
| Belgium | 1 (7.7%) |
| Netherlands | 1 (7.7%) |
| **Medical specialty** |  |
| Nephrology | 1 (7.7%) |
| Neurology | 9 (69.2%) |
| Neuropaediatric dep. | 1 (7.7%) |
| Metabolic specialists | 2 (15.4%) |
| **Type of centre the HCP works in:** |  |
| Specialised Unit without Gene Therapies experience | 5 (38.5%) |
| Specialised Unit with Gene Therapies experience | 8 (61.5%) |

HCP: Healthcare provider.

### Recommendations/guidelines

#### European Union (EU) guidelines

Most HCPs (69.2%) reported being unaware of the EU recommendations, suggesting a widespread knowledge gap and limited access to these guidelines. Respondents were stratified by prior experience with gene therapy (**Supplementary Appendix Figure 1**). Only 25% of HCPs working in units with experience in gene therapy were aware of EU guidelines (predominantly referring to the guidelines on the follow-up of patients who had received gene therapy medicinal products). In addition, 40% of respondents with no prior experience in gene therapy were aware of EU guidelines. These HCPs mainly referred to recommendations provided by the EMA. However, their responses also highlighted the limited availability of gene therapy in their clinical settings. For instance, gene therapy was noted to be primarily accessible in paediatric hospitals, with specific reimbursement criteria (spinal muscular atrophy [SMA]: reimbursement <6 months old, with some availability for SMA children <13.5 kg, if older, criteria defined) restricting its use.

Supplementary Appendix Figure 1 Prior experience of HCPs with European gene therapy guidelines

GT: Gene therapy; HCPs: Healthcare providers.

#### National guidelines

Awareness of national guidelines for gene therapy among HCPs was notably low, with 84.6% of respondents reporting unfamiliarity with these recommendations. Only 15.4% were aware, primarily from Luxembourg and Germany, specialised in neuropaediatrics and nephrology, and mainly used recommendations from the EMA guidelines and the guidelines for the follow-up of patients who were administered gene therapy medicinal products.

#### Local/institutional guidelines

Most of the HCPs (61.5%) reported being unaware of the local/institutional recommendations. Among HCPs with prior experience in gene therapy, 62.5% were aware of local or institutional guidelines, often implemented through pharmacy standard operating procedures (SOPs) or institutional protocols (**Supplementary Appendix Figure 2**). Regarding the review process, these recommendations or SOPs were updated every six months, annually, or remained unspecified, depending on the country. The remaining 37.5%, working in Denmark, Hungary, and Germany, were unaware of local guidelines. All HCPs without gene therapy experience (100%) reported no awareness of local guidelines. These professionals were based in Greece, the Netherlands, Austria, Poland, and Luxembourg and specialised in neurology, metabolic disorders, and nephrology.

Supplementary Appendix Figure 2 Prior experience of HCPs with local/institutional gene therapy guidelines

HCPs: Healthcare providers.

### Gene therapy treatment administration

Most treatments were managed by neuropaediatricians, accounting for 42.9% of respondents, followed by neurologists (28.5%) (**Supplementary Appendix Table 2**).

Supplementary Appendix Table 2 Specialty of gene therapy treatment administered

| **Who administers gene therapy treatment in your department?** | **Nº of HCPs (%)(n=13)^a^** |
| --- | --- |
| Neurology | 4 (28.5%) |
| Neuropaediatric | 6 (42.9%) |
| Haematology | 1 (7.15%) |
| Oncology | 1 (7.15%) |
| Metabolic specialists | 1 (7.15%) |
| Not specified | 1 (7.15%) |

^a^ One HCP specified both neurology and neuropaediatric. HCP: Healthcare provider.

Most cases were followed by neuropaediatricians (35.7%) and neurologists (28.5%) (**Supplementary Appendix Table 3**).

Supplementary Appendix Table 3 Specialty of gene therapy follow-up responsible

| **Who does the gene therapy follow-up in your department?** | **Nº of HCPs (%)(n=13)^a^** |
| --- | --- |
| Neurology | 4 (28.5%) |
| Neuropaediatric | 5 (35.7%) |
| Haematology | 2 (14.3%) |
| Metabolic specialists | 1 (7.15%) |
| Paediatric dep. | 1 (7.15%) |
| Not specified | 1 (7.15%) |

^a^ One HCP specified both neurology and neuropaediatric. HCP: Healthcare provider.

Most of the HCPs responsible for gene therapy care were neurologists or neuropaediatricians, collectively accounting for 71.4% of the responses (**Supplementary Appendix Table 4**).

Supplementary Appendix Table 4 Specialty of gene therapy care responsible

| **Who is taking care of gene therapy in your department?** | **Nº of HCPs (%)(n=13)^a^** |
| --- | --- |
| Neurology | 5 (35.7%) |
| Neuropaediatric | 5 (35.7%) |
| Haematology | 1 (7.15%) |
| Metabolic specialists | 1 (7.15%) |
| Paediatric dep. | 1 (7.15%) |
| Not specified | 1 (7.15%) |

^a^ One HCP specified both neurology and neuropaediatric. HCP: Healthcare provider.

Gene therapy treatment administration by countries is shown in **Supplementary Appendix Table 5.**

Supplementary Appendix Table 5 Summary of gene therapy personnel responsible for administration, follow-up, and care among countries

| **Country** | **Who administers gene therapy treatment in your department?** | **Who does the gene therapy follow-up in your department?** | **Who is taking care of gene therapy in your department?** |
| --- | --- | --- | --- |
| **Hungary** | Neuropaediatric dep | Neuropaediatric dep | Neuropaediatric dep |
| **Austria** | Haematology | Haematology | Haematology |
| **Belgium** | Neurology | Neurology | Neurology |
| **Denmark** | Neurology | Neurology | Neurology |
| **France** | Neuropaediatric dep | Paediatric dep | Paediatric dep |
| **Germany** | Metabolic specialists | Metabolic specialists | Metabolic specialists |
| **Germany** | Neuropaediatric dep | Neuropaediatric dep | Neuropaediatric dep |
| **Germany** | Neuropaediatric dep | Neuropaediatric dep | Neuropaediatric dep |
| **Greece** | Neurology | Neurology | Neurology |
| **Italy** | Adult and paediatric neurologist | Adult and paediatric neurologists | Adult and paediatric neurologists |
| **Luxembourg** | Oncology | Haematology | Neurology |
| **Netherlands** | It is not specified | It is not specified | It is not specified |
| **Poland** | Neuropaediatric dep | Neuropaediatric dep | Neuropaediatric dep |

Dep: Department.

### Gene therapy trials

Around half (53.8%) of the HCPs were participating in gene therapy trials. Of those actively involved in trials, 42.8% were involved in two trials, 28.6% in one trial, and 28.6% in three trials. HCPs participating in one trial were based in Belgium and Germany and specialised in neurology and neuropaediatrics. Those involved in two trials were from France, Italy, and Germany, including metabolic specialists and neurologists. Finally, HCPs participating in three trials were in Denmark and Luxembourg and specialised in nephrology and neurology.

### Gene therapy prescription

Almost half (46.2%) of the respondents routinely prescribed gene therapy. Of those who routinely prescribed GT, 83.3% treated more than ten patients, whereas 16.7% treated more than five. The HCPs managing over ten patients were in Greece, Hungary, Poland, Germany, and Italy, and were specialised in neurology and neuropaediatrics, with a combination of those with and without prior gene therapy experience. Meanwhile, the HCP treating over five patients was based in Luxembourg, specialised in nephrology, and had no previous GT experience.

### Final comments

As part of the survey, participants were asked to share any comments, suggestions, or documents they believed might be relevant.

Supplementary Appendix Table 6 : Final comments, suggestions, and other relevant information.

| **Country** | **Medical Specialty** | **Type of centre you work in:** | **Please share any comments or suggestions that you think may be of interest** |
| --- | --- | --- | --- |
| Greece | Neurology | Without GT experience | Just to note that in my centre, we administer gene therapies (ASOs) for SMA patients, and we adhere to the guidelines applicable to this condition. |
| Hungary | Neurology | With GT experience | A European *in vivo* GT guideline would be important. I am aware only of FDA guidelines for industry. |
| Poland | Neurology | Without GT experience | Gene therapy seems to be the future for many neuromuscular diseases. In my country, gene therapy is currently available to SMA patients only. |
| Germany | Neuropaediatric dep. | With GT experience | Support for application of gene therapy and monitoring of patients is important. |
| Italy | Neurology | With GT experience | So far, only ASO-based therapy is available for adults; for children, we use both ASO- and AAV-based therapies. |
| Luxembourg | Nephrology | Without GT experience | - |
| Netherlands | Neurology | Without GT experience | - |
| Austria | Metabolic specialists | Without GT experience | - |
| Germany | Neurology | With GT experience | - |
| France | Neurology | With GT experience | Gene therapy trials currently concern more paediatric units than neurology units. |
| Belgium | Neurology | With GT experience | I only considered gene therapy at DNA level, not RNA level; if this is wrong, I will have to redo the survey. |
| Germany | Metabolic specialists | With GT experience | The issues regarding gene therapy are very disease- and therapy-specific. |
| Denmark | Neurology | With GT experience | This is too extensive. I would have many comments, but highly dependent on which type of gene therapy. |

AAV: Adeno-associated virus; ASO: Antisense oligonucleotides; FDA: United States Food and Drug Administration; GT: Gene therapy; SMA: Spinal muscular atrophy.
